# Unmet need for family planning and its associated factor among women of reproductive age in Debre Berhan Town, Amhara, Ethiopia

**DOI:** 10.1186/s13104-019-4180-9

**Published:** 2019-03-15

**Authors:** Solomon Adanew Worku, Sindew Mahmud Ahmed, Tizebt Fisseha Mulushewa

**Affiliations:** 1 Department of Midwifery, College of Health Science, Debre Berhan University, Debre Berhan, Ethiopia; 2 Departments of Nursing, College of Health Science, Debre Berhan University, Debre Berhan, Ethiopia

**Keywords:** Family planning, Limiting, Spacing, Unmet need

## Abstract

**Objective:**

Unmet need refers to fecund women who either wish to postpone the next birth (spacers) or who wish to stop childbearing (limiters) but are not using a contraceptive method. The aim of this study was to assess the unmet needs of family planning and identify associated factors in Debre Berhan town among women in reproductive age. The community-based cross-sectional study design was used among 411 study participants (women with reproductive age) at Debre Berhan town. A systematic sampling technique was used to select the households. Bivariate and multivariable analyses were done to determine the association of each independent variable with the dependent variable.

**Results:**

The overall unmet need for family planning among women in reproductive age groups was found to be 30.9%. Occupational status AOR = 13.992 (1.054–185.833), from whom the respondents got information about family planning AOR = 0.018 (0.002–0.170), having a discussion with husband AOR = 16.692 (2.911–95.713) and support from husband AOR = 0.005 (0.001–0.025) was significantly associated with the outcome variable. The level of unmet need for family planning in the study area is still high compared to the target set (10%) in the national family planning guide plan for Ethiopia.

## Introduction

Unmet need refers to fecund women who either wish to postpone the next birth (spacers) or who wish to stop childbearing (limiters) but are not using a contraceptive method [1, 2]. Many women who are sexually active would prefer to avoid becoming pregnant but are not using any method of contraception. These women are considered to have an unmet need for family planning [3].

Globally an estimated 80 million unintended pregnancies, both mistimed and unwanted, occur each year. Unintended pregnancy and births have grave consequences to the mother and family and are global social and health burdens. It is much more likely to end in potentially unsafe abortion [4].

Sub-Saharan Africa, 25 percent of women of reproductive age who are married or in a union have an unmet need for family planning. Also, four countries in Latin America and the Caribbean, eight countries in Asia and four countries in Oceania have an unmet need for family planning above 20 percent according to the most recent data available [5, 6].

Report from EDHS 2016 reveals that 58% of currently married women age 15–49 have a demand for family planning. Thirty-six percent of currently married women are already using a contraceptive method either to space (22%) or to limit births (14%). Unmet need for currently married women age 15–49 is lowest in Addis Ababa (11%) and highest in Oromia region (29%) [7].

Unmet need has a direct impact on the total fertility rate. It is believed that if unmet need were eliminated, fertility would decline substantially. From a policy perspective, reducing unmet need for family planning is important for both achieving demographic goals and enhancing individual rights [8].

To reduce the proportion of unmet need for family planning, knowing the current level and its determinants is a prerequisite. This study was conducted to investigate the magnitude and associated factors of unmet need for FP among reproductive-age women in Debre Berhan town.

## Main text

### Study design, study population and sampling

The study was conducted from March to April 2018 in Debre Berhan town which is located in north shoa zone of Amhara regional state of Ethiopia. The town is located 130 km away from the capital city of Addis Ababa on the North East direction along the main road to Wollo and Tigray [9]. The community-based cross-sectional study design was employed. All women’s age 15–49 in the selected sample Kebeles were our study population. To determine the number of women to be included in the study a single population proportion formula was used. The final sample size was 411 [2]. In this study unmet need for family planning refers to fecund women who either wish to postpone the next birth or who wish to stop child bearing but are not using a contraceptive method. A systematic sampling technique was used to select the households (HH), and a woman in reproductive age in the selected households was interviewed.

### Data collection and data analysis

Data were collected by trained data collectors. Both open and close-ended semi-structured interview administered questionnaire was utilized for data collection. The questionnaire was developed in English based on literature review [2, 10–12] and translated into Amharic, then back to English to check for consistency. Finally, the Amharic version was used for data collection.

The data was intensively cleaned up before its analysis and was entered using Epi Data 3.1 version and analysis was carried out using statistical package for social sciences (SPSS) version22. Frequency distribution tables and statistical graphs were used to describe some variables. Cross-tabulation and logistic regression were done to examine the association between dependent and independent variables and significant variables (p-value less than 0.2) were entered into multivariate analysis and adjusted odds ratio (AOR) was seen to check confounding factors. A 95% confidence level and a p-value of less than 0.05 were considered to get statistically significant.

### Results

#### Socio-demographic characteristics

A total of 411 women were enrolled in this study with a response rate of 100%. Their mean age was found to be 29.4 ± 7.65 years. The majority of them were orthodox in religion 333 (81.0%). In terms of marital status 238 (57.9%) married and Housewives 120 (29.2%). About 200 (48.7%) of the respondents were complete college and above. Almost all of the respondents 395 (96.1%) had access to mass media (Table 1).

**Table 1:** Socio demographic characteristics of the respondents in Debre Berhan town April 2018 N = 411

Variable	Response	Frequency	Percent
Age of respondents N = 411 Mean age = 29.4 years	≤ 25	147	35.8
	26–35	158	38.4
	36–45	98	23.8
	≥ 46	8	2
Religion of the respondents N = 411	Orthodox	333	81.0
	Protestant	41	10.0
	Muslim	29	7.1
	Catholic	5	1.2
	Other	3	0.7
Marital status of the respondents N = 411	Single	147	35.8
	Married	238	57.9
	Separated	9	2.2
	Widowed	17	4.1
Educational status of the respondents N = 411	Can’t read and write	19	4.6
	Can read and write	41	10.0
	Primary school	58	14.1
	Secondary school	93	22.6
	College and above	200	48.7
Occupational status of respondents N = 411	House wife	120	2 9.2
	Student	66	16.1
	Employed	167	40.6
	S Self-employed	58	14.1
Husband education level N = 238	Can’t read and write	5	2.1
	Can read and write	9	3.8
	Primary school	33	13.8
	Secondary school	53	22.3
	College and above’	138	58.0
Access to mass media n = 411	Yes	395	96.1
	No	16	3.9

#### Reproductive history

From the total of respondents, 276 (67.2%) of the participants were experienced pregnancy in their lifetime. From those who experience pregnancy 85 (30.8%) of them experience pregnancy one times. Majority of the respondents 245 (88.8%) gave birth. About 58 (21%) of respondents who were experienced pregnancy had a history of abortion. Twenty-seven (6.5%) of the interviewed women were pregnant and all of the pregnancy was wanted. Majority of the respondents 398 (95.6%) of had information about family planning. From those who had got information about family planning 155 (39.4%) of the respondents got from health workers and had got a discussion about how to use family planning from health providers 193 (91.9%). From those who don’t use family planning the major reason raised by the respondents was fear of side effect, 56 (27.9%) and least majority doesn’t utilize family planning because they want to have more children.

#### Unmet need for family planning

**Fig. 1:**
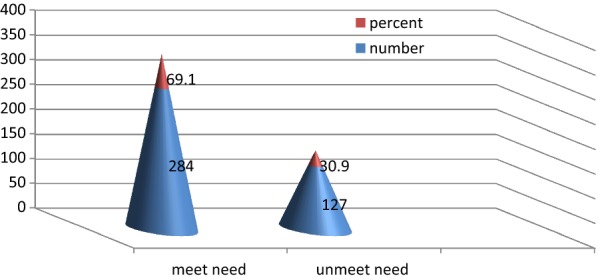
Respondents over all unmet need for family planning among women in reproductive age group in Debre Berhan Town April 2018 (n = 411)

Unmet need was calculated by summing the number of women who do not use family planning as a result fear of side effects, prohibition from husband, prohibition from religion and inaccessibility. The overall unmet need for family planning among women in reproductive age groups was found to be 30.9% (Fig. 1).

#### Factors affecting the unmet need for family planning

After controlling other variables among the independent variables only, occupational status, a place from where heard about family planning, had a discussion with husband and got support from your husband to use family planning maintained their association with unmet need for family planning after controlling other confounders. A respondent whose occupation is employed was found to be 13.992 times more likely to had an unmet need for family planning compared with self-employed.AOR = 13.992 (1.054–185.833)*.

Women who heard information about family planning from health worker 98.2% less likely to had an unmet need for family planning compared with those who got from community AOR = 0.018 (0.002–0.170)***. The respondent who had a discussion with their husband about family planning 16.692 times more likely to had an unmet need for family planning compare with those who had no discussion with their husbands AOR = 16.692 (2.911–95.713)**.

Respondents who got support from husband 99.5% less likely to had an unmet need for family planning compared with who had no support. AOR = 0.005 (0.001–0.025)** (Table 2).

**Table 2:** Association between independent variables and unmet need for family is planning among women in the reproductive age group in Debre Berhan Town April 2018

Variables	Alternative response	Unmet need planning		COR (95% CI)	AOR (95% CI)	p-value
		Yes	No			
Occupation	Housewife	36	84	2.333 (1.037–5.250)	6.751 (.485–93.938)	0.155
	Student	33	33	5.444 (2.306–12.854)	68.108 (0.478–9697.946)	0.095
	Employed	49	118	2.261 (1.031–4.957)	13.992 (1.054–185.833)*	0.046
	Self-employed	9	49	1.00	1.00	
From where do you hear	From mass media	48	97	0.2830 (0.111–0.720)	0.185 (0.030–1.142)	0.069
	From health worker	22	133	0.095 (0.036–0.252)	0.018 (0.002–0.170)***	≤ 0.001
	From school	21	26	0.462 (0.163–1. 308)	0.044 (0.001–1.141)	0.115
	From friends	8	15	0.305 (0.090–1.033	0.267 (0.028–2.516)	0.249
	From husband	1	0	923,128,494.031	7,447,944,062.228	1
	From community	14	8	1.00	1.00	
Have you discussed family planning with your husband	Yes	38	170	0.540 (0.253–1.254)	16.692 (2.911–95.713)**	0.02
	No	12	29	1.00	1.00	
Have you got support from your husband to use family planning	Yes	7	162	0.032 (0.013–0.079)*	0.005 (0.001–0.025)**	≤ 0.001
	No	40	30	1.00	1.00	

* Significant variables

### Discussion

The present study intended to calculate the unmet need for family planning in Debre Berhan Town. The overall unmet need was found to be 30.9% which was much higher than the national prevalence which was 22% of currently married women have an unmet need for family planning [7]. This variation is due to the EDHS includes only married women. But ours include married and unmarried women. And higher in the study conducted in Mekele which was 21.4%, the study in Dangela (17.4%) [2, 11, 13]. The variation may be due to the difference in study subjects in which those studies include only married reproductive age groups but, our include all reproductive age groups. It is also higher the study in Kishanganj district, Bihar, India which was 23.9% [14]. This variation is may be due to the difference in Socio-demographic characteristic of the study subjects.

The finding of the study was low compared with the study conducted in Sudan Using West off model the total unmet need was estimated at 44.8%, in Arba Minch (41.5%) [2, 10]. The variation may be due to the difference in sample size as well as socioeconomic characteristics of the study participants.

Our study reveals that a respondent whose occupation is employed was found to be 13.992 times more likely to had an unmet need for family planning compared with self-employed. This finding is higher than the study done in Dangla that reveals occupationally women who are none employed found to be 6.81 times more likely to had unmet need compared with respondents who was employed [11]. The result of our study is different from the study in Dangla due to the difference in sample size and Socio-demographic characteristics of respondents or this are due to employed women may have no time and busy by work.

The present study reveals that respondents who got support from husband 99.5% less likely to had an unmet need for family planning compared with who had no support. This is supported by the study conducted in Pakistan and Areba Minch [2, 15]. This study revealed that women whose partner had a non-supportive attitude about contraceptives use were more likely to have an unmet need for family planning compared to women whose partners had a supportive attitude.

The present study reveals that respondent who had a discussion with their husband about family planning 16.692 times less likely to have an unmet need for family planning compare with those who had no discussion with their husbands. This is inlined with the study conducted in Nigeria [16]. This implies that decisions around family size and fertility will enhance family planning utilization. The present study also reveals that there is a significant relationship between unmet need for family planning and from whom respondents heard information for family planning. It indicates that Women who heard information about family planning from health worker 98.2% less likely to had an unmet need of family planning compared with those who got from community This was not discussed with other studies. And it reflects that discussion with health provider will enhance family planning utilization and decrease unmet need.

Our study reveals that there was no significant association between unmet need for family planning and the number of lived children. But studies in Kenya, Areba Minch, Sudan & Uganda reveals that the likelihood of having unmet need seemed to increase with the number of living children. Couples who have more living children are more likely to have an unmet need than the ones who have fewer children or none at all. Couples with more children have a greater desire to stop childbearing, which may not be translated into actual practice, because of other factors affecting the decision to use family planning, or those that affect the supply and accessibility of family planning [2, 10, 17, 18]. In our case, it may be due to an inadequate sample size that only a small portion of the respondents had living children.

In this study even if they are not significant Husband’s disapproval, prohibition from religion and fear of side effects were found to be the common reasons behind the unmet need for family planning among the respondents of the present study. This is supported by the studies in Pakistan, Sudan, Uganda and Bangladesh [4, 10, 15, 17]. The study in Saudi Arabia reveals that major reason why women do not go in for use of a contraceptive despite their desire to do so revealed lack of knowledge and lack of access to family planning methods, fear of side effect of contraceptives, religious prohibition, and husband’s disapproval as commonly stated reasons [18].

### Conclusions

The study reveals that there was a high prevalence of unmet need in the study area 30.9% and family planning utilization rate was found to be 51.1%. Family planning among reproductive-age women was not met. Variables like occupation, support from the husband, from who heard about family planning and had a discussion with husband about family planning have a significant association with unmet need.

### Recommendation

It is important to strengthen counseling and partner involvement in Debre Berhan Town to reduce unmet need for family.

### Limitations

The men were not included as participants to understand their perception towards the total unmet need for family planning. The study did not asses separately unmet need for spacing or limiting because the tool doesn’t have a parameter to assess these variables.

## Abbreviations

AOR: Adjusted Odds Ratio
EDHS: Ethiopia Demographic and Health Survey
HH: households
SPSS: statistical package for social science
